# The EMPOWER Occupational e–Mental Health Intervention Implementation Checklist to Foster e–Mental Health Interventions in the Workplace: Development Study

**DOI:** 10.2196/48504

**Published:** 2024-03-15

**Authors:** Alberto Raggi, Renaldo M Bernard, Claudia Toppo, Carla Sabariego, Luis Salvador Carulla, Sue Lukersmith, Leona Hakkaart-van Roijen, Dorota Merecz-Kot, Beatriz Olaya, Rodrigo Antunes Lima, Desirée Gutiérrez-Marín, Ellen Vorstenbosch, Chiara Curatoli, Martina Cacciatore

**Affiliations:** 1 Neurology, Public Health and Disability Unit Fondazione IRCCS Istituto Neurologico Carlo Besta Milano Italy; 2 Swiss Paraplegic Research Nottwil Switzerland; 3 Faculty of Health Sciences and Medicine University of Lucerne Lucerne Switzerland; 4 Center for Rehabilitation in Global Health Systems University of Lucerne Lucerne Switzerland; 5 Health Research Institute University of Canberra Canberra Australia; 6 Healthcare Information Systems (CTS553) University of Cadiz Cadiz Spain; 7 Erasmus School of Health Policy and Management Erasmus University Rotterdam Netherlands; 8 Institute of Psychology University of Lodz Lodz Poland; 9 Research, Innovation and Teaching Unit Parc Sanitari Sant Joan de Déu Sant Boi de Llobregat Spain; 10 Centro de Investigación Biomédica en Red de Salud Mental (CIBERSAM) Madrid Spain

**Keywords:** implementation, workplace, mental health, well-being, digital health, mobile health, mHealth, eHealth, e–mental health, stakeholder consultation, intervention, occupational, stakeholders, consultation, barrier, checklist

## Abstract

**Background:**

Occupational e–mental health (OeMH) interventions significantly reduce the burden of mental health conditions. The successful implementation of OeMH interventions is influenced by many implementation strategies, barriers, and facilitators across contexts, which, however, are not systematically tracked. One of the reasons is that international consensus on documenting and reporting the implementation of OeMH interventions is lacking. There is a need for practical guidance on the key factors influencing the implementation of interventions that organizations should consider. Stakeholder consultations secure a valuable source of information about these key strategies, barriers, and facilitators that are relevant to successful implementation of OeMH interventions.

**Objective:**

The objective of this study was to develop a brief checklist to guide the implementation of OeMH interventions.

**Methods:**

Based on the results of a recently published systematic review, we drafted a comprehensive checklist with a wide set of strategies, barriers, and facilitators that were identified as relevant for the implementation of OeMH interventions. We then used a 2-stage stakeholder consultation process to refine the draft checklist to a brief and practical checklist comprising key implementation factors. In the first stage, stakeholders evaluated the relevance and feasibility of items on the draft checklist using a web-based survey. The list of items comprised 12 facilitators presented as statements addressing “elements that positively affect implementation” and 17 barriers presented as statements addressing “concerns toward implementation.” If a strategy was deemed relevant, respondents were asked to rate it using a 4-point Likert scale ranging from “very difficult to implement” to “very easy to implement.” In the second stage, stakeholders were interviewed to elaborate on the most relevant barriers and facilitators shortlisted from the first stage. The interview mostly focused on the relevance and priority of strategies and factors affecting OeMH intervention implementation. In the interview, the stakeholders’ responses to the open survey’s questions were further explored. The final checklist included strategies ranked as relevant and feasible and the most relevant facilitators and barriers, which were endorsed during either the survey or the interviews.

**Results:**

In total, 26 stakeholders completed the web-based survey (response rate=24.8%) and 4 stakeholders participated in individual interviews. The OeMH intervention implementation checklist comprised 28 items, including 9 (32.1%) strategies, 8 (28.6%) barriers, and 11 (39.3%) facilitators. There was widespread agreement between findings from the survey and interviews, the most outstanding exception being the idea of proposing OeMH interventions as benefits for employees.

**Conclusions:**

Through our 2-stage stakeholder consultation, we developed a brief checklist that provides organizations with a guide for the implementation of OeMH interventions. Future research should empirically validate the effectiveness and usefulness of the checklist.

## Introduction

### Background

Mental health is a major concern in the workplace and is considered a worldwide public health priority. In Europe, stress, depression, and anxiety are the third-most common mental health problems caused or worsened by work [1]. About half of the European workers report stress and psychosocial risks as a common phenomenon in the workplace, causing approximately 50% of all working days lost [2]. Of the working-age population, 6%-8% of females and 4.0%-5.5% of males suffer from depression and 5%-6% of females and around 3% of males suffer from anxiety [3]. Depression and anxiety are common mental disorders that impact the working status of individuals in terms of unemployment, absenteeism (ie, loss of workdays due to a condition), and presenteeism (ie, reduced ability at work due to a condition). A review study showed that 21% of unemployed people have depression and that depression and unemployment are strongly interrelated [4]. In addition, research shows that past and current anxiety or depressive disorders are associated with higher absenteeism, with the highest effect in those with comorbid anxiety and depression [5]. This has economic consequences: mental ill health was estimated to cost the world economy about US $2.5 trillion a year in 2010, with a projection of US $6 trillion by 2030 [6]. Investment in mental health promotion is needed, and it is estimated that each dollar invested in scaled-up treatment for depression and anxiety will produce a US $4 return on investment in improved health and productivity [6].

A growing body of evidence identifies psychosocial and contextual risk factors related to mental health at the workplace, such as working conditions (eg, job insecurity, work-life balance, job pressure), organizational culture, and type of work [7-9]. For instance, anxiety and barriers at the workplace, including social, attitudinal, and health system–related environmental ones, are key determinants of work performance for workers with depression [10]. Moreover, other factors, such as highly conflicting and excessive work demands, time pressure, low autonomy levels, excessive authority, and lack of social support within the workplace, can increase stress and the risk of developing or worsening mental health conditions [11-13]. These workplace-based factors are critical as good mental health in the workplace is important for the well-being and success of employees, employers, and society.

Addressing mental health issues in the workplace ideally serves a dual purpose: to enhance the well-being of both employees and employers and to strengthen employers’ ability to manage stressful situations. In this regard, existing evidence on occupational e–mental health (OeMH) interventions indicates their effectiveness in promoting mental health and alleviating the associated burdens [14-16]. In particular, a meta-analysis by Carolan et al [15] showed that workplace mental health interventions delivered via mobile technologies, computers, or the internet are effective in promoting psychological well-being and reducing anxiety and depression. Although evidence on their effectiveness has been exhaustively evaluated, research addressing implementation strategies, barriers, and facilitators is limited [17-19]. A contributing factor is the lack of guidance for documenting and reporting successful implementation of OeMH interventions, as well as related barriers and facilitators [17].

Although limited, implementation research on OeMH interventions has revealed a myriad of strategies, barriers, and facilitators that influence the successful implementation of these interventions [17]. A brief and user-friendly checklist comprising key implementation factors is potentially instrumental, if used prior to implementation, in ensuring the successful uptake of OeMH interventions. Such a checklist would be particularly useful for those who are interested in implementing OeMH interventions, including those who develop interventions, the employers, and the implementation team.

To develop such a checklist, the knowledge and perspective of stakeholders are crucial. Developers of OeMH interventions typically focus on the technical and content-related aspects of the interventions. The available research on the strategies for OeMH intervention implementation and particularly implementation in the workplace is limited [17]. Furthermore, we could not identify research, based on a real-wold experience, that accounted and systematically analyzed the knowledge and opinion of end users and of other organizational stakeholders on factors that should be considered for OeMH intervention implementation. Stakeholder consultations regarding the uptake of interventions are essential during the phases of development, implementation, and evaluation of interventions in the workplace [20]. The experience-based knowledge and expertise gained from stakeholders can provide unique insights into the major factors that facilitate or hinder implementation in a workplace setting [21].

### Objective

The objective of this study was to develop a brief checklist including the most relevant and feasible strategies, barriers, and facilitators for the implementation of OeMH interventions. We achieved this objective through 2 different stages: first, by reducing a comprehensive set of strategies, barriers, and facilitators, compiled in a recently published systematic review [17], through an online survey that involved a set of relevant stakeholders and, second, by refining the information through individual interviews with a restricted number of stakeholders who were available to provide additional feedback.

## Methods

### Overview

We carried out a 2-stage stakeholder consultation as part of the EMPOWER (European Platform to Promote Wellbeing and Health in the workplace) project; a more detailed description of the project can be found in the EMPOWER protocol paper [22].

As part of the EMPOWER project activity, we contacted a group of stakeholders through contacts from EMPOWER consortium collaborators, and additional possible contacts were provided by the already contacted stakeholders. A total of 120 stakeholders were included on our list: representatives from advocacy groups, labor organizations, government organizations, nongovernmental organizations (NGOs), the scientific community, and other interested parties, including the European Commission, universities, vocational training institutions, employment advisors, and members of the European Parliament, specifically those participating in the interparliamentarian group on disability and mental health. The 120 stakeholders from our list were predominantly female (n=71, 59.2%) and on average 50 years old. Presumably, they had on average 20 years of experience; 44 (36.7%) worked for NGOs or policy institutions and 30 (25%) were from related research areas. In most cases (n=68, 56.7%), they had a managerial or executive role within their organizations. Additionally, we contacted 25 senior authors of papers included in the scoping review by Bernard et al [17] to complement the practice-level perspective with a more technical perspective of those who had implemented an OeMH intervention and described strategies, barriers, and facilitators in a publication.

### Stakeholder Consultation

#### First Stage: Web-Based Survey

The first consultation stage involved participants completing a web-based survey in English, implemented using Google Forms. The survey included a brief section requesting sociodemographic information, such as country of employment, highest career position, work sector, and organization type and size (based on the Organization for Economic Co-operation and Development [OECD] classification: microenterprises for <10 employees, small enterprises for 10-49 employees, medium-size enterprises for 50-249 employees, and large enterprises for 250 or more employees) [23]. Respondents were asked to rate the relevance and feasibility of items on a comprehensive checklist comprising strategies, barriers, and facilitators relevant to OeMH intervention implementation identified in the scoping review by Bernard et al [17]: specifically, 13 strategies for the implementation of OeMH interventions, 12 associated facilitators, and 17 barriers were included in the survey. A detailed description of the online survey is included in Multimedia Appendix 1.

Questions about the implementation strategies addressed the relevance and feasibility of each strategy operationalized in terms of ease or difficulty of practical implementation. For each strategy, the first response option was “not relevant,” which was meant to exclude the strategy from further consideration. If a strategy was deemed relevant, respondents were asked to rate it using a 4-point Likert scale ranging from “very difficult to implement” to “very easy to implement.” The facilitators and barriers were presented to participants as statements addressing “elements that positively affect implementation” and “concerns toward implementation,” respectively. Participants were asked to indicate the extent to which they agreed with the statements on a 5-point Likert scale ranging from “strongly disagree” to “strongly agree,” with a neutral middle position. The survey also included optional open-ended questions where participants could elaborate on responses and suggest additional implementation strategies, barriers, or facilitators not mentioned in the closed-ended questions. Responses were collected between January 31 and March 31, 2022, and reminder emails to complete the survey were sent every 2 weeks during this time.

#### Second Stage: Semistructured Individual Interviews

Qualitative semistructured individual interviews were conducted with stakeholders who agreed on participating in a follow-up interview after the web-based survey or in the interview only. An interview guide was developed based on the survey results:

- Stakeholder background, including the field of work and level of experience
- General comments on the survey’s results (showed to the interviewee during the interview)
- Analysis of the relevance and priority of strategies and factors affecting OeMH intervention implementation
- Analysis of responses to the survey’s open questions

The interviews were held online and in English, led by a senior researcher and assisted by 2 researchers, who took notes. A summary of the interview transcript was sent to interviewees for approval after each interview.

### Ethical Considerations

Prior to the consultation, participants were asked to read and provide informed consent. The protocol for the consultation was approved by the Ethics Committee of the Fundació Sant Joan de Déu (ref: PS-19-20). Participation was on a voluntary basis: no compensation was offered.

The 120 stakeholders included on our list already provided consent to being contacted again for research purposes at the time they were included on our list. The 25 senior authors of the papers included in Bernard et al’s [17] review were contacted using the same approach we used for the stakeholders on the list, the only difference being that they were directly informed that the survey was online.

The information produced from the web-based survey was anonymous (ie, no survey field with names and contact information was mandatory). Therefore, to be contacted also for the interview, participants willing to contribute had to explicitly disclose their identities.

### Data Analysis

#### First Stage: Web-Based Survey

Microsoft Excel was used for calculations. We used frequency-based percentages to report how strategies were perceived by respondents. We ranked the strategies from the easiest to the most difficult to implement based on the response options (very easy, easy, difficult, and very difficult); strategies were defined as easy to implement if ≥50% of the respondents reported them as easy or very easy and difficult to implement if ≥50% of the respondents reported them as difficult or very difficult. Frequency-based percentages were also used to report the level of agreement among respondents regarding the identification of barriers and facilitators. We ranked the barriers and facilitators based on the degree of agreement, from the most agreed ones (ie, those with higher percentages of “strongly agree” and “agree”) to the less agreed ones (ie, those with higher percentages of “strongly disagree” and “disagree”). Barriers and facilitators were considered relevant to our analysis if ≥50% of the respondents agreed with the particular item.

#### Second Stage: Semistructured Individual Interviews

A qualitative description was provided. We used the notes taken during interviews to (1) address whether strategies, barriers, and facilitators not endorsed from the survey were instead endorsed by the interviewees; (2) address whether open-ended survey questions might suggest a new construct, which should be included in the final checklist, or whether it was a different formulation of an item included in the comprehensive list of strategies, barriers, and facilitators; and (3) define additional strategies, barriers, and facilitators. Factors identified during the interviews, and not previously raised, were then retained for inclusion in the OeMH intervention implementation checklist.

### EMPOWER OeMH Intervention Implementation Checklist Development

The final EMPOWER OeMH intervention implementation checklist was developed considering the 2-stage consultation process. Strategies were retained if ≥50% of the respondents judged them as “easy” or “very easy” to implement, whereas barriers and facilitators were retained if ≥50% of the respondents agreed with considering an item as either a barrier or a facilitator, respectively. In addition, we retained those items that did not reach the 50% threshold but that were endorsed during the individual interviews, as well as those added by interviewees and not included in the survey (and thus not identified by the literature review). Therefore, the checklist included strategies ranked as relevant and feasible and the most relevant facilitators and barriers. To improve the user-friendliness of the checklist, we rephrased all items so that users can check whether a condition is met or avoided (in the case of barriers).

## Results

### First Stage of the Stakeholder Consultation: Web-Based Survey

#### Descriptive Statistics

Of the 145 individuals to whom emails were sent, 105 (72.4%) participants were reached as 40 (27.6%) emails were inactive. Of those 105 participants reached, 26 (24.8%) completed the survey. They reported being from Poland (n=4, 15%), Finland (n=4, 15%), Spain (n=3, 12%), Switzerland (n=2, 8%), and Malta (n=2, 8%). The remaining 42% (n=11) were originally from other European countries (Slovenia, Austria, Belgium, Germany, Serbia, Lithuania, the United Kingdom, the Czech Republic, Croatia, Sweden, and Denmark). Half of the stakeholders worked in health and social work activities, 26.9% (n=7) in education, 15.4% (n=4) in the field of research, 3.8% (n=1) in the information and communication sector, and 3.8% (n=1) in a patient NGO. Most worked mainly in the public sector (n=22, 84.6%). Some (n=7, 26.9%) represented senior management, 26.9% (n=7) middle management, 23.1% (n=6) executive management (eg, chief executive officer [CEO]), 15.4% (n=4) operations (eg, staff), and 7.7% (n=2) a consultant position. At the time of the survey, 65.4% (n=17) worked in a large enterprise, 15.4% (n=4) in a medium-size one, and 19.2% (n=5) in a microenterprise or a small enterprise (ie up to 49 employees).

#### Implementation Strategies

**Figure 1:**
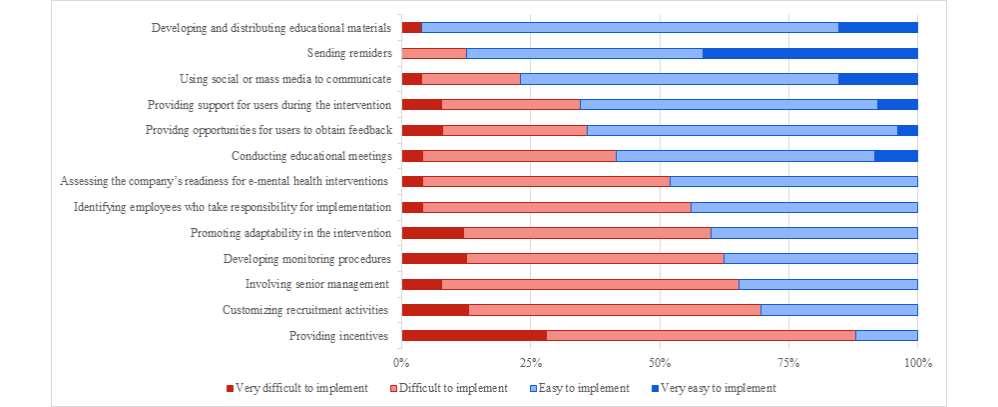
Frequency-based ranking of strategies’ easiness of implementation.

Almost all 13 implementation strategies were rated as relevant (see Multimedia Appendix 2 for frequency tables). The strategy rated as havin the least relevance was “customizing recruitment activities to enhance reach” (n=3, 12%). Figure 1 shows the frequency-based ranking of the strategies.

In addition, 6 (46.2%) implementation strategies were identified as “easy to implement” or “very easy to implement” by ≥50% of the respondents: “developing and distributing education material,” “sending reminders for completing the intervention,” “using social or mass media to increase reach,” “providing support for users during the intervention,” “providing opportunities for users to obtain feedback on progress,” and “conducting educational meetings.” Conversely, “involving senior management,” “customizing recruitment,” and “providing incentives” were perceived as the most difficult to implement.

#### Barriers and Facilitators

**Figure 2:**
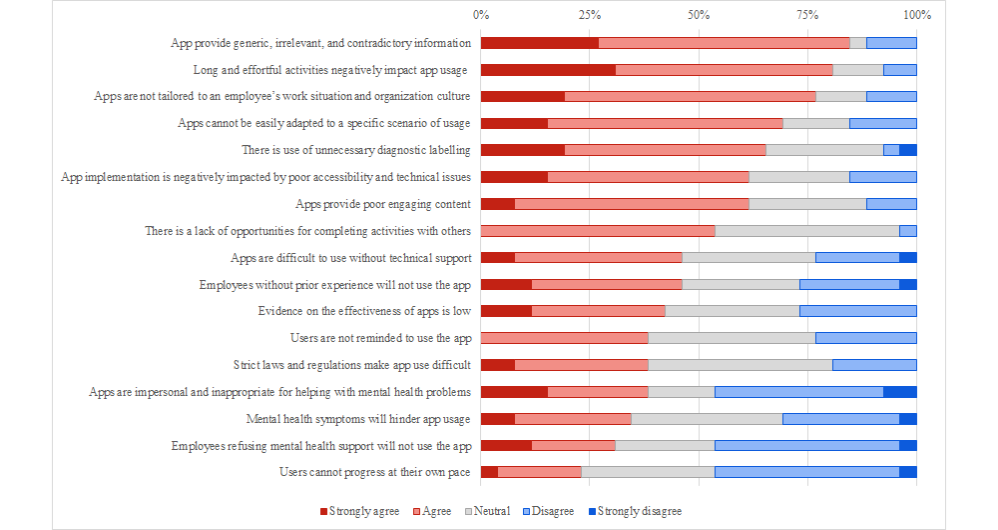
Frequency-based ranking of barriers.

Of the 17 elements presented as potential barriers towards implementation, 8 (47.1%) were considered as such by participants. Specifically, the most negatively perceived barriers were “apps providing generic, irrelevant, or contradictory information,” “long and effortful activities negatively impact usage of apps,” and “apps are not tailored to an employee’s situation and organization’s culture.” Conversely, 9 (52.9%) barriers were not endorsed by participants: in particular, the less negatively perceived barriers were “mental health symptoms will hinder app usage,” “employees refusing mental health support will not use the app,” and “users cannot progress at their own pace.” Figure 2 shows the frequency-based ranking of the barriers.

**Figure 3:**
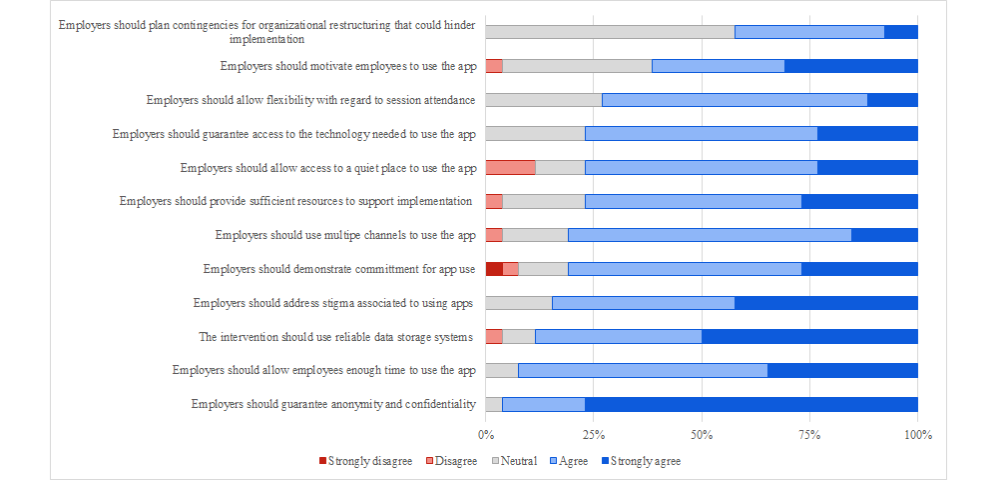
Frequency-based ranking of facilitators.

All the 12 items presented as potential facilitators toward implementation were perceived as such, with the exception of “employers should plan contingencies for organizational restructuring that could hinder implementation.” Among the endorsed facilitators, the 3 (27.3%) rated as most relevant were “employers should guarantee anonymity and confidentiality,” “employers should allow employees enough time to use the app,” and “the intervention should use reliable data storage systems.” Figure 3 shows the frequency-based ranking of the facilitators.

Overall, the responses to the web-based survey showed greater heterogeneity in the evaluations of barriers, whereas a general agreement toward facilitators was observed. Specifically, the key elements identified included the importance of the intervention’s usability, the ease of access and use, the presence of engaging content, the exclusion of excessively effortful activities, and the presence of accurate information without diagnostic labeling.

#### Optional Open-Ended Questions

In addition to the predefined list of strategies, barriers, and facilitators, stakeholders provided some additional suggestions in optional open-ended questions included in the web-based survey, including 7 strategies, 3 barriers, and 3 facilitators (see Textbox 1).

**Additional strategies, facilitators, and barriers suggested in the first consultation stage (web-based survey), provided by the 26 participants who completed the web-survey.**

Strategies

- Encouraging dialogue with trade unions and workers
- Providing access to a helpline
- Involving occupational health specialists
- Conducting market analysis to identify similar apps and address posed risks to the successful implementation of the newly proposed app
- Involving people with experience of mental disorders who are already using a similar tool
- Conducting usability tests before launch to verify the app’s actual usability and, if required, improving it before its full implementation
- Testing the occupational e–mental health (OeMH) intervention with health managers (eg, from the human resources departments) of large companies

Barriers

- Noninvolvement of end users in cocreation
- Confidentiality issues (eg, will others, such as my boss or colleagues, know that I am using such an app?)
- Unclear data protection policies that poorly convey who, especially within the organization, can view usage information

Facilitators

- Having an open dialogue with managers showing willingness to share information about their past problems with mental health difficulties in the workplace (eg, stress or anxiety)
- Clearly identifying who is in the position of deciding whether to use the app (ie, the employer or single employees?)
- Providing mental health interventions in established mental health and community services

### Second Stage of the Stakeholder Consultation: Semistructured Individual Interviews

A total of 4 stakeholders, 2 (50%) from Italy, 1 (25%) from Serbia, and 1 (25%) from Denmark, agreed to participate in an individual interview. These stakeholders came from different work sectors, namely human resources, disability management, and health education and public health, respectively. During the interviews, 5 topics were related to implementation strategies and 4 to barriers and facilitators.

#### Main Interview Topics on Implementation Strategies

Five main topics related to implementation strategies were addressed during the interviews, and 1 new strategy was suggested by an interviewee.

First, the assessment of an organization’s readiness to implement OeMH interventions was considered a crucial preliminary step to be taken regardless of its difficulty.

Second, strategies exclusively focused on the simple “distribution of information” were deemed at a high risk of failure due to being too impersonal and because they cannot be used alone. Examples of these included “developing and distributing education material,” “sending reminders for completing the intervention,” and “using social or mass media to increase reach.” These strategies could be perceived as ways employers provide quick mental health solutions that are inadequately personalized. One interviewee described such kind of information-focused strategies as “a fit for all approach that may not fit anyone.”

Third, a similar consideration was made about strategies aimed at involving occupational health specialists and senior management (eg, sharing of experience). Such strategies cannot work if used alone. On the one hand, they might be effective for fostering the promotion of interventions; on the other hand, much of these strategies’ success depends on the “personality” of the managers (ie, the degree to which they are available to “get involved” also on a personal level), rather than the overall culture of specific workplaces, regardless of whether such kind of personal exposure is realistic.

Fourth, involving employees (ie, the end users) in the implementation processes is a necessary strategy to avoid them perceiving interventions as being imposed by management. In fact, the level of adherence to an intervention might be seriously hindered if the intervention itself is perceived as mandatory.

Fifth, a new strategy was suggested by an interviewee, which involved offering interventions as an employee benefit provided by the company. This would have the advantage of employees seeing the intervention as an investment for their welfare. Further, it might be particularly valuable for those working in small and medium-size enterprises, where health and social interventions may be not available through the workplace, in contrast to those working in large enterprises.

#### Main Interview Topics on Barriers and Facilitators

Four main topics focused on barriers and facilitators: none of them constituted a new topic, and they were a further endorsement of topics already included in the survey.

First, interviewees stressed that using unnecessary diagnostic labels within interventions (eg, marketing it as “a way to combat depression”) might hinder their acceptance.

Second, interviewees highlighted the risk associated with the stigma surrounding the use of OeMH interventions, a topic that in the web-based survey was included among facilitators (ie, “Employers should address the stigma associated with app use.”). The role of employers in combating stigma associated with app use was, of course, emphasized.

Third, interviewees stressed the importance of employers as guarantors of anonymity and confidentiality of any information that is shared when using the intervention.

Fourth, it is important that employers demonstrate their commitment to adopting mental health interventions, while motivating employees to use such interventions and maintaining a dialogue with them.

### EMPOWER OeMH Intervention Implementation Checklist

There was widespread agreement between findings from the survey and interviews, except regarding the level of management participation and the idea of proposing the OeMH intervention as a benefit for employees. By combining the results of Bernard et al’s [17] scoping review and this stakeholder consultation, we developed an EMPOWER OeMH intervention implementation checklist (Textbox 2). It comprises 28 items: 9 (32.1%) address implementation strategies, 8 (28.6%) refer to implementation barriers, and 11 (39.3%) refer to facilitators for implementation.

**The EMPOWER (European Platform to Promote Wellbeing and Health in the workplace) occupational e–mental health (OeMH) intervention implementation checklist.**

Implementation strategies (n=9)

- Promotion messages are sent via mass media channels to reach a large audience.
- Educational material has been developed and distributed (eg, leaflets or emails).
- Educational meetings are planned or have been conducted.
- Organization readiness has been assessed before intervention implementation.
- The intervention is proposed as a benefit for employees.
- End users have been involved throughout the implementation process.
- Support for using the intervention (eg, online support for technical problems with the app or platform) is available.
- Opportunities for users to obtain feedback on progress (eg, percentage of completion) are available.
- Reminders for completing the intervention are envisaged.

Barriers (n=8)

- Avoid generic, irrelevant, contradictory, or inaccurately translated content.
- Avoid long and effortful tasks.
- Avoid interventions that are not tailored to employees’ work situation and the organization’s culture.
- Avoid intervention components that exclusively target specific use cases and are difficult to adapt to other scenarios.
- Avoid unnecessary diagnostic labeling in the intervention (eg, people with depression or stress).
- Avoid poor accessibility, technical issues, and complicated user interfaces.
- Avoid content that is not engaging and presented in a single media format.
- Avoid having all tasks be single-user tasks (ie, no group activities).

Facilitators (n=11)

- Guarantee anonymity and confidentiality.
- Allow employees enough time to use the intervention.
- Use reliable data storage systems for the intervention.
- Address any stigma associated with using an OeMH intervention.
- Demonstrate the employers’ commitment to and interest in employees’ participation in the intervention .
- Use multiple communication channels (eg, leaflets, emails, or meetings) to promote the intervention.
- Provide sufficient resources (eg, money or personnel) to support the implementation of the intervention.
- Provide a quiet and private space for employees to use the intervention.
- Ensure access to the technology required (eg, devices or WiFi) to use the intervention.
- Allow flexibility regarding intervention attendance during work hours.
- Motivate employees to use the intervention.

## Discussion

### Principal Findings and Comparison With Prior Work

We developed an EMPOWER OeMH intervention implementation checklist, a 28-item checklist including 9 strategies, 8 barriers, and 11 facilitators, which guides the implementation of OeMH interventions. The checklist constitutes a step forward in documenting different implementation strategies for OeMH interventions and might fill the gap in the understanding of the reasons some OeMH interventions succeed and others fail.

Two major difficulties were identified in the implementation of OeMH interventions: first, involving the actors of the organizations, particularly those in management roles, in the promotion of the intervention and, second, adapting and customizing the intervention to the individual organization and end-user needs. The 3 strategies evaluated as most relevant and easy to implement (namely, “developing and distributing education material,” “sending reminders,” and “using social or mass media to increase reach”) include simple activities in terms of the time needed to implement them and are expected to easily reach more end users, in line with a previous study [24]. Unsurprisingly, such automated and less time-consuming strategies were considered easier to implement. As shown in a recent systematic review [25], automated strategies foster a positive user experience and offer a low-cost alternative to human support. On the contrary, our interviewees issued a warning about the use of automated strategies as they could be perceived as impersonal, which may result in a low level of commitment from employers. For these reasons, we support a multiplicity of strategies, not just automated ones.

A low level of commitment is a disadvantage: if employees think that employers are uncommitted, the likelihood of benefiting from the OeMH intervention will be much lower. Our results highlight the importance of organizational commitment in promoting an OeMH intervention, including the need to identify and dedicate resources to the implementation, such as giving employees flexibility, time, and space to participate in the intervention. These findings are in line with previous research showing that the lack of allocated time and adequate private space could hinder engagement with these interventions [25,26]. Organizational commitment is also relevant to addressing stigma, which is known to be a significant barrier to the implementation of mental health services in the community [27]. In contrast, antistigma interventions at the workplace are overwhelmingly effective [28]. Therefore, addressing stigma, including cultural factors that perpetuate stigma attached to mental illness [29], could be critical to foster the implementation of OeMH interventions.

Usability is an essential feature to enhance user engagement, as outlined in different studies on mental health interventions [25,30,31]. This is in line with the recommendations of the “Use of Digital Technology” guidelines [32-34], which advocate for enhancing content clarity through the use of explicit labels and sentences to describe content and instructions in the OeMH interventions.

The interview and web-based survey results were closely aligned on barriers and facilitators, whereas a different perspective on implementation strategies was offered by interviewees. A key point was the need to actively involve both management and end users in the process of implementation in order to avoid perceiving OeMH interventions as mandatory. The involvement of all actors, in particular end users, is a general principle in our checklist, recognized through one of the items. Of course, involvement has a voluntary basis, as no one can be forced to engage in any kind of intervention: from the side of companies, this means providing all possible facilitators that enable use and involvement in OeMH intervention uptake in the workplace. Such a general principle is thus recognized in the last facilitators included in our checklist, which focus on resources and space provision; access to technologies, if needed; time flexibility; and fostering of employees’ motivation. The survey findings about the inclusion of senior management, judged as less feasible, and about preferring strategies that are time limited apparently contradict the interview results, which pointed out that an initial investment of time, including time from managers, might be preferred to foster the uptake of OeMH interventions. The latter is in line with previous work suggesting the importance of management participation in the early stages of implementation in order to make strategic decisions that meet the specific needs of organizations [26], thus positively influencing intervention success [35,36].

Assessing organizational readiness before implementing an intervention is a crucial step [37-40], and it was judged among the most relevant strategies by the interviewees. Such a readiness assessment should consider any stigma attached to mental illness within the organization and ensure the anonymity and data protection of end users. Organizations failing to account for these issues carry the risk that OeMH interventions will be negatively perceived by employees (eg, seen as an attempt to deceive employees or to force them to reveal their mental health status or as an untrustworthy system incapable of keeping employee health information private and secure).

Interviewees suggested presenting OeMH interventions as an economic benefit for employees. Highlighting both a personal and an economic value conveys the message that organizations invest in improving employees’ health and well-being, exactly in the way in which other services, either health related or otherwise, are presented as part of their remuneration. Such a strategy was deemed to be potentially effective as it demonstrates a strong commitment from companies. Indeed, effectively communicating the intervention’s proven benefits for employee end users is an important strategy for uptake [26].

A due consideration is that no single strategy exists to manage the complex process of OeMH intervention implementation. The strategies that have been identified because of the different stages of our activity are summative and highly complementary: the possibility to use a combined set of different approaches, composed of different strategies and facilitators, in the process of OeMH intervention implementation is therefore the preferred approach. The kind of OeMH intervention, the cultural context (including the organization’s culture and values as well as the country’s culture), and the circumstance in which the OeMH intervention is proposed (eg, response to a particular event or as a more general approach for promoting employees’ mental health) may influence implementation.

### Implications and Recommendations for Practice and Future Research

The EMPOWER OeMH intervention implementation checklist is a good starting point to determine the factors that enhance the success of OeMH interventions. Implementation research on OeMH interventions can also benefit from our checklist to collect information about the most usfeful strategies and about the most important barriers to and facilitators of implementation. More research is needed on the general validity, usefulness, and acceptability of different implementation strategies, as well as a broadly accepted definition of successful implementation (eg, number of users, usage over time, outcomes).

### Limitations

The main limitation of this study is related to the small stakeholder sample size. A total of 26 stakeholders responded to the online survey, corresponding to a response rate of 24.8% and a limited number of them participated in the individual interviews. Such a low response rate is likely due to staff turnover of people who held institutional positions. Although a common problem for web-based surveys, studies with our observed ~25% response rate have demonstrated consistent and accurate results [41,42]. Although the invitation was sent to several stakeholders from different geographical areas, it has to be remembered that potential respondents were limited to those referred by EMPOWER consortium members and to the senior authors of the papers included in Bernard et al. [17] review. Participants had heterogeneous backgrounds and position levels within the respective organizations, which was an advantage as it likely led to obtaining a wide and heterogeneous set of items. Results may have been different, in terms of the amount of additional implementation strategies, barriers, or facilitators suggested during this study, if there was a larger group of stakeholders. A second limitation is that the checklist has not been piloted and evaluated in an implementation project. Future research is thus needed to validate the checklist.

### Conclusion

We developed an EMPOWER OeMH intervention implementation checklist comprising key strategies, barriers, and facilitators relevant to the implementation of OeMH interventions. The checklist could serve as a guide for organizations implementing OeMH interventions and those reporting on these implementations. Future studies that implement this checklist and assess its validity, usefulness, and acceptability in different contexts should be planned.

## Appendix

Multimedia Appendix 1 Web survey form.

Multimedia Appendix 2 Frequency tables for implementation strategies, barriers, and facilitators.

## Abbreviations

EMPOWER: European Platform to Promote Wellbeing and Health in the workplace
OeMH: occupational e–mental health
NGO: nongovernmental organization

## Data Availability

The deidentified quantitative data sets generated and analyzed during the study are available from the corresponding author upon reasonable request as long as the main objective of the data-sharing request is to replicate the analysis and findings, as reported in this paper (without investigator support), after approval of a proposal and with a signed data access agreement. The qualitative data sets generated and analyzed during the study are not publicly available due the risk of reidentification of participants.
