# Supplementary material for: The EMPOWER Occupational e–Mental Health Intervention Implementation Checklist to Foster e–Mental Health Interventions in the Workplace: Development Study
Source: J Med Internet Res. 2024 Mar 15;26:e48504. doi: 10.2196/48504 (PMC10980995; doi:10.2196/48504)
Supplement: Multimedia Appendix 1 [file jmir_v26i1e48504_app1.docx]

**Title:** The EMPOWER Occupational eMental Health implementation checklist: A tool for fostering eMental Health interventions in the workplace

**Authors:** Alberto Raggi, Renaldo M. Bernard, Claudia Toppo, Carla Sabariego, Luis Salvador Carulla, Sue Lukermith, Leona Hakkaart-van Roijen, Dorota Merecz-Kot, Beatriz Olaya Guzman, Rodrigo Antunes Lima, Desirée Gutierrez, Ellen Vorstenbosch, Chiara Curatoli, Martina Cacciatore

**Multimedia Appendix 1: Web survey form**

**Context information**

Before starting the questionnaire, we would like to ask you for some information that will help us contextualise and therefore better understand your responses. The questions below refer to the most important work activity you have carried out in your life.

1. In which country have you predominantly been working?

________________________

2. In which sector have you predominantly been working?

1. Private
2. Public

3. In which industry have you predominantly been working?

1. Health and social work activities
2. Information and communication
3. Financial and insurance services
4. Education
5. Hospitality and leisure
6. Utilities – electricity gas
7. Manufacturing
8. Construction
9. Real estate activities
10. Retail
11. Other: __________________

4. How large is the company you have worked at?

1. Small company (i.e. 1 to 49 employees)
2. Medium-sized company (i.e. 50 to 249 employees)
3. Large company (i.e. 250 or more employees)

5. What is the highest position you have held in your career?

1. Executive management (e.g. CEO)
2. Senior management
3. Middle management
4. Operations (e.g. staff)
5. Consultant

**Implementation strategies**

Below there are 13 implementation strategies that involve activities that focus on different aspects of implementing an eMental health intervention, like the EMPOWER platform. We ask you to indicate the feasibility of each implementation strategy. If you think that the strategy is not relevant, please mark only the option “not relevant”.

In doing so, please imagine that a company, like the one you have been working for, wishes to implement an eMental health intervention to improve employees’ well-being and tackle mental health problems.

1. Assessing the company’s readiness for eMental health interventions and tailoring strategies to address identified barriers (e.g. identify the needs, preferences, and interests of stakeholder groups that influence usage and delivery)

1. NOT RELEVANT
2. Very difficult to implement
3. Difficult to implement
4. Easy to implement
5. Very easy to implement

2. Identifying employees who take responsibility for implementing the intervention (e.g. organisational champions who dedicate time to promoting and supporting the implementation of the intervention)

1. NOT RELEVANT
2. Very difficult to implement
3. Difficult to implement
4. Easy to implement
5. Very easy to implement

3. Involving senior management (e.g. encouraging management to introduce, publicly support, and tailor implementation activities)

1. NOT RELEVANT
2. Very difficult to implement
3. Difficult to implement
4. Easy to implement
5. Very easy to implement

4. Conducting educational meetings (e.g. senior and middle management conduct introductory seminars with employees aimed at explaining the intervention, securing acceptance, providing answers to questions, and inspiring participation)

1. NOT RELEVANT
2. Very difficult to implement
3. Difficult to implement
4. Easy to implement
5. Very easy to implement

5. Developing and distributing educational materials (e.g. send materials on the benefits of using the eMental health intervention)

1. NOT RELEVANT
2. Very difficult to implement
3. Difficult to implement
4. Easy to implement
5. Very easy to implement

6. Using social or mass media to communicate with a large audience and increase reach (e.g. recruiting users via email, organisational intranet or leaflets, social media, internet newspaper, TV, and radio)

1. NOT RELEVANT
2. Very difficult to implement
3. Difficult to implement
4. Easy to implement
5. Very easy to implement

7. Customising recruitment activities to enhance reach (e.g. offer the possibility to get the eMental health intervention through a direct contact with high-level managers)

1. NOT RELEVANT
2. Very difficult to implement
3. Difficult to implement
4. Easy to implement
5. Very easy to implement

8. Promoting adaptability in the intervention to meet each organisation’s needs (e.g. ensuring the intervention is flexible enough to be easily offered to employees with different levels of education, with different job profiles and possibly in different languages)

1. NOT RELEVANT
2. Very difficult to implement
3. Difficult to implement
4. Easy to implement
5. Very easy to implement

9. Sending reminders for completing the intervention (e.g. sending automated email or in-app notifications based on user-determined intervals and user activity)

1. NOT RELEVANT
2. Very difficult to implement
3. Difficult to implement
4. Easy to implement
5. Very easy to implement

10. Providing support for users during the intervention (e.g. provide email support and sufficient in-app instructions for users to use the app as intended)

1. NOT RELEVANT
2. Very difficult to implement
3. Difficult to implement
4. Easy to implement
5. Very easy to implement

11. Providing incentives (e.g. participation in the intervention is rewarded with free hours off work or with economic incentives)

1. NOT RELEVANT
2. Very difficult to implement
3. Difficult to implement
4. Easy to implement
5. Very easy to implement

12. Providing opportunities for users to obtain feedback on progress (e.g. participants receive automated and tailored feedback, and can monitor their activity over time)

1. NOT RELEVANT
2. Very difficult to implement
3. Difficult to implement
4. Easy to implement
5. Very easy to implement

13. Developing monitoring procedures that enable to address the successful implementation of the intervention (e. g. conducting meetings finalized to address the effectiveness of the implementation strategy, i.e. monitoring the amount of app users)

1. NOT RELEVANT
2. Very difficult to implement
3. Difficult to implement
4. Easy to implement
5. Very easy to implement

14. If any, what additional strategies should be considered for the successful implementation of a mobile app aimed at preventing and reducing the impact of mental health problems at the workplace?

___________________________

**Concerns regarding implementation**

People who implement eMental health apps in organisations highlight several concerns regarding implementation, some of which are listed below. Please indicate how much you agree or disagree with each statement.

15. I am concerned that the implementation of eMental health apps is negatively impacted by poor accessibility, technical issues, and complicated user interfaces

1. Strongly disagree
2. Disagree
3. Neutral
4. Agree
5. Strongly agree

16. I am concerned that eMental health apps are difficult to use independently without support (e.g. technical support)

1. Strongly disagree
2. Disagree
3. Neutral
4. Agree
5. Strongly agree

17. I am concerned that the inclusion of long and effortful activities (e.g. long surveys) negatively impact usage of eMental health apps

1. Strongly disagree
2. Disagree
3. Neutral
4. Agree
5. Strongly agree

18. I am concerned that opportunities for completing activities in eMental health apps with others (e.g. group activities) are sometimes not provided

1. Strongly disagree
2. Disagree
3. Neutral
4. Agree
5. Strongly agree

19. I am concerned about eMental health apps that are not tailored to an employee’s work situation and organisation’s culture

1. Strongly disagree
2. Disagree
3. Neutral
4. Agree
5. Strongly agree

20. I am concerned about eMental health apps that cannot be easily adapted to a specific scenario of usage

1. Strongly disagree
2. Disagree
3. Neutral
4. Agree
5. Strongly agree

21. I am concerned that users cannot progress through the app at their own pace

1. Strongly disagree
2. Disagree
3. Neutral
4. Agree
5. Strongly agree

22. I am concerned that users are sometimes not reminded (e.g. notifications) or given feedback (e.g. progress tracking) that encourages them to use the app

1. Strongly disagree
2. Disagree
3. Neutral
4. Agree
5. Strongly agree

23. I am concerned that strict laws and regulations make using innovative eMental health apps difficult

1. Strongly disagree
2. Disagree
3. Neutral
4. Agree
5. Strongly agree

24. I am concerned about the evidence for the effectiveness of an eMental health app

1. Strongly disagree
2. Disagree
3. Neutral
4. Agree
5. Strongly agree

25. I am concerned about the use of unnecessary diagnostic labelling (e.g. ‘depressed people’) in eMental health apps

1. Strongly disagree
2. Disagree
3. Neutral
4. Agree
5. Strongly agree

26. I am concerned about apps providing generic, irrelevant, contradictory, and inaccurately translated information

1. Strongly disagree
2. Disagree
3. Neutral
4. Agree
5. Strongly agree

27. I am concerned about apps providing insufficiently engaging content that is unavailable in multiple formats (e.g. audio, video)

1. Strongly disagree
2. Disagree
3. Neutral
4. Agree
5. Strongly agree

28. I am concerned that eMental health apps are impersonal (e.g. lacking human interaction) and inappropriate tools for helping with sensitive topics such as mental health problems

1. Strongly disagree
2. Disagree
3. Neutral
4. Agree
5. Strongly agree

29. I am concerned that symptoms of mental health conditions will hinder app usage

1. Strongly disagree
2. Disagree
3. Neutral
4. Agree
5. Strongly agree

30. I am concerned that employees are not willing to seek mental health support and will not use eMental health apps

1. Strongly disagree
2. Disagree
3. Neutral
4. Agree
5. Strongly agree

31. I am concerned that employees without prior experience will not use eMental health apps

1. Strongly disagree
2. Disagree
3. Neutral
4. Agree
5. Strongly agree

32. If any, what additional concerns should be considered for the successful implementation of a mobile app aimed at preventing and reducing the impact of mental health problems at the workplace?

___________________________

**Elements that positively affect implementation**

People who implement eMental health apps in organisations also highlight several elements that positively affect implementation, some of which are listed below. Please indicate how much you agree or disagree with each statement.

33. Employers should demonstrate a commitment for the usage of the app by employees

1. Strongly disagree
2. Disagree
3. Neutral
4. Agree
5. Strongly Agree

34. Employers should provide sufficient resources (e.g. money) to support implementation

1. Strongly disagree
2. Disagree
3. Neutral
4. Agree
5. Strongly Agree

35. Employers should guarantee anonymity and confidentiality (e.g. using a privacy policy)

1. Strongly disagree
2. Disagree
3. Neutral
4. Agree
5. Strongly Agree

36. Employers should allow employees enough time to use the app

1. Strongly disagree
2. Disagree
3. Neutral
4. Agree
5. Strongly Agree

37. Employers should allow employees access to a quiet and private space to complete app exercises

1. Strongly disagree
2. Disagree
3. Neutral
4. Agree
5. Strongly Agree

38. Employers should motivate employees to use eMental health apps

1. Strongly disagree
2. Disagree
3. Neutral
4. Agree
5. Strongly Agree

39. Employers should address any stigma associated with using an eMental health app

1. Strongly disagree
2. Disagree
3. Neutral
4. Agree
5. Strongly Agree

40. Employers should plan contingencies for organisational restructuring (e.g. turnover) that could hinder the implementation

1. Strongly disagree
2. Disagree
3. Neutral
4. Agree
5. Strongly Agree

41. The intervention should use reliable data storage systems (i.e. reduces the risk of data loss)

1. Strongly disagree
2. Disagree
3. Neutral
4. Agree
5. Strongly Agree

42. Employers should use multiple channels (e.g. intranet and posters) to promote the app

1. Strongly disagree
2. Disagree
3. Neutral
4. Agree
5. Strongly Agree

43. Employers should ensure employees access to the technology (e.g. smartphone, tablet) required to use the app

1. Strongly disagree
2. Disagree
3. Neutral
4. Agree
5. Strongly Agree

44. Employers should allow flexibility with regard to session attendance (e.g. recording live sessions should be available)

1. Strongly disagree
2. Disagree
3. Neutral
4. Agree
5. Strongly Agree

45. If any, what additional positive elements should be considered for the successful implementation of a mobile app aimed at preventing and reducing the impact of mental health problems at the workplace?

___________________________

**Thanks!**

You have reached the end of the questionnaire. We greatly appreciate your time and valuable contribution!

**Credits for contribution**

If you would like to be formally cited as a consultant in our technical report for the European Commission, please add your name, affiliated institution, and country below.

First name ___________________________

Last name ___________________________

Institution, Country ___________________________

**Individual interview**

If you wish to participate in an online interview of around 20 minutes, please enter your email address below and we will contact you shortly. Interviewing you will be especially important if you provided additional suggestions for implementation strategies, concerns, or positive elements.

Name ___________________________

Email ___________________________
