# Supplementary material for: The EMPOWER Occupational e–Mental Health Intervention Implementation Checklist to Foster e–Mental Health Interventions in the Workplace: Development Study
Source: J Med Internet Res. 2024 Mar 15;26:e48504. doi: 10.2196/48504 (PMC10980995; doi:10.2196/48504)
Supplement: Multimedia Appendix 2 [file jmir_v26i1e48504_app2.docx]

**Title:** The EMPOWER Occupational eMental Health implementation checklist: A tool for fostering eMental Health interventions in the workplace

**Authors:** Alberto Raggi, Renaldo M. Bernard, Claudia Toppo, Carla Sabariego, Luis Salvador Carulla, Sue Lukermith, Leona Hakkaart-van Roijen, Dorota Merecz-Kot, Beatriz Olaya Guzman, Rodrigo Antunes Lima, Desirée Gutierrez, Ellen Vorstenbosch, Chiara Curatoli, Martina Cacciatore

**Multimedia Appendix 2: Frequency tables for implementation strategies, barriers and facilitators**

**Table S1.** *Items’ scores distribution for each implementation strategy.*

|  | Not Relevant | Very Difficult to implement | Difficult to implement | Easy to implement | Very easy to implement |
| --- | --- | --- | --- | --- | --- |
| Developing and distributing educational materials | 0 | 1 | 0 | 21 | 4 |
| Sending reminders | 2 | 0 | 3 | 11 | 10 |
| Using social or mass media to communicate | 0 | 1 | 5 | 16 | 4 |
| Providing support for users during the intervention | 0 | 2 | 7 | 15 | 2 |
| Providing opportunities for users to obtain feedbacks | 1 | 2 | 7 | 15 | 1 |
| Conducting educational meetings | 2 | 1 | 9 | 12 | 2 |
| Assessing the company’s readiness for eMental health interventions | 1 | 1 | 12 | 12 | 0 |
| Identifying employees who take responsibility for implementation | 1 | 1 | 13 | 11 | 0 |
| Promoting adaptability in the intervention | 1 | 3 | 12 | 10 | 0 |
| Developing monitoring procedures | 2 | 3 | 12 | 9 | 0 |
| Involving senior management | 0 | 2 | 15 | 9 | 0 |
| Customizing recruitment activities | 3 | 3 | 13 | 7 | 0 |
| Providing incentives | 1 | 7 | 15 | 3 | 0 |

**Table S2.** *Items’ scores distribution for each barrier.*

|  | Strongly Agree | Agree | Neutral | Disagree | Strongly Disagree |
| --- | --- | --- | --- | --- | --- |
| Apps provide generic, irrelevant and contradictory information | 7 | 15 | 1 | 3 | 0 |
| Long and effortful activities negatively impact apps usage | 8 | 13 | 3 | 2 | 0 |
| Apps are not tailored to an employee’s work situation and organization culture | 5 | 15 | 3 | 3 | 0 |
| Apps cannot be easily adapted to a specific scenario of usage | 4 | 14 | 4 | 4 | 0 |
| Use of unnecessary diagnostic labelling | 5 | 12 | 7 | 1 | 1 |
| App implementation is negatively impacted by poor accessibility and technical issues | 4 | 12 | 6 | 4 | 0 |
| Apps providing poor engaging content | 2 | 14 | 7 | 3 | 0 |
| Lack of opportunities for completing activities with others | 0 | 14 | 11 | 1 | 0 |
| Apps are difficult to use without technical support | 2 | 10 | 8 | 5 | 1 |
| Employees without prior experience will not use the app | 3 | 9 | 7 | 6 | 1 |
| Evidence on the effectiveness of apps is low | 3 | 8 | 8 | 7 | 0 |
| Users are not reminded to use the app | 0 | 10 | 10 | 6 | 0 |
| Strict laws and regulations make apps use difficult | 2 | 8 | 11 | 5 | 0 |
| Apps are impersonal and inappropriate for helping with mental health problems | 4 | 6 | 4 | 10 | 2 |
| Mental health symptoms will hinder app usage | 2 | 7 | 9 | 7 | 1 |
| Employees refusing mental health support will not use the app | 3 | 5 | 6 | 11 | 1 |
| Users cannot progress at their own pace | 1 | 5 | 8 | 11 | 1 |

**Table S3.** *Items’ scores distribution for each facilitator.*

|  | Strongly Disagree | Disagree | Neutral | Agree | Strongly Agree |
| --- | --- | --- | --- | --- | --- |
| Employers should guarantee anonymity and confidentiality | 0 | 0 | 1 | 5 | 20 |
| Employers should allow employees enough time to use the app | 0 | 0 | 2 | 15 | 9 |
| The intervention should use reliable data storage systems | 0 | 1 | 2 | 10 | 13 |
| Employers should address stigma associated to using apps | 0 | 0 | 4 | 11 | 11 |
| Employers should demonstrate commitment for app use | 1 | 1 | 3 | 14 | 7 |
| Employers should use multiple channels to use the app | 0 | 1 | 4 | 17 | 4 |
| Employers should provide sufficient resources to support implementation | 0 | 1 | 5 | 13 | 7 |
| Employers should allow access to a quiet place to use the app | 0 | 3 | 3 | 14 | 6 |
| Employers should guarantee access to the technology needed to use the app | 0 | 0 | 6 | 14 | 6 |
| Employers should allow flexibility with regard to session attendance | 0 | 0 | 7 | 16 | 3 |
| Employers should motivate employees to use the app | 0 | 1 | 9 | 8 | 8 |
| Employers should plan contingencies for organizational restructuring that could hinder implementation | 0 | 0 | 15 | 9 | 2 |
